# Speaking Up Against Hierarchy: A Simulation Geared Towards Nursing Students

**DOI:** 10.7759/cureus.11977

**Published:** 2020-12-08

**Authors:** Celina Da Silva, Eva Peisachovich, Charles K Anyinam, Sue Coffey, Leslie Graham, Farideh Tavangar

**Affiliations:** 1 Medical Education and Simulation, York University, Toronto, CAN; 2 Simulation, Nipissing University, Toronto, CAN; 3 Nursing, Ontario Tech University, Toronto, CAN; 4 Medical Education and Simulation, Durham College, Toronto, CAN; 5 Nursing, York University, Toronto, CAN

**Keywords:** conflict management, randomized controlled trial, pre-simulation activity, communication skills, revised medical research council framework, simulation education

## Abstract

Background

As simulation science continues to advance, the focus previously put on scenario creation and debriefing must now be applied to other components of the learning experience. There is a need to examine the effectiveness of pre-simulation activities and how they relate to the overall simulation experience and learning outcomes. However, few randomized controlled trials have been conducted comparing different approaches in the pre-simulation preparatory phase and the impact on learning outcomes.

Methods

A randomized controlled trial was conducted with undergraduate nursing students (n=83) who were randomized to a traditional paper case study (control group) or an interactive pre-simulation activity (intervention group). The use of the two-challenge rule and Satisfaction and Self Confidence in Learning (SSL) was evaluated.

Results

The proportion of students who utilized the two-challenge rule in the intervention group was significantly higher than the control group. Results from the two independent-samples Wilcoxon-Mann-Whitney test showed a significant difference in the median of the total score of the SSL W=2.5, p <0.001, satisfaction W=6.0, p <0.001, and self-confidence W=68.0, p <0.001 in learning between third-year nursing students in the control and intervention groups.

Conclusion

Our results showed significant differences in the use of the two-challenge rule by students who completed an interactive pre-simulation activity (intervention group) compared to those who completed the paper case study (control group). Additionally, students in the intervention group were more self-confident and satisfied with the entire simulation intervention than the control group. From a pedagogical perspective, this study also emphasizes the need to ground simulations in theory. Moreover, there is value in using progressive frameworks, i.e., revised Medical Research Council (2014) in simulation design and research to ensure high quality. More studies are required to examine the right dosage and type of pre-simulation activity and impact on learning outcomes.

## Introduction

Conflict is a clash or struggle occurring when a real or perceived threat or difference exists in the desires, thoughts, attitudes, feelings, or behaviours of two or more individuals [1]. Communication is central to how conflict is productively or destructively managed [2,3]. Health care teams experience interpersonal conflict involving two or more people, possibly related to specific tasks, social interactions, or process-related issues [4,5,6].

Nursing students begin to experience interpersonal conflict when they enter clinical placements with preceptors and other nurses that, if unchecked, can lead to unsafe care, including medication errors [4,6,7,8]. One method of preparing nursing students for clinical placements is utilizing high-fidelity simulations representing specific aspects of nursing care realistically. There is limited research to determine the use of high-fidelity simulation to improve undergraduate nursing students’ conflict management skills [7,9,10]. Moreover, more experimental studies are required to evaluate the pre-simulation preparation component of simulations and its’ impact on learning outcomes [11]. For this study, a pre-simulation activity is a preparatory work performed before attending a high-fidelity simulation. Pre-simulation preparation involves students engaging in lectures, readings, quizzes, videos, and PowerPoint slides.

The objective of this randomized controlled trial (RCT) study was to examine the effectiveness of two types of pre-simulation activities on learner outcomes. Undergraduate, third-year nursing students were randomized to either 1) a traditional paper case study (control group) or 2) a video simulation case study (intervention group) before participating in a high-fidelity simulation. The learning outcomes were a) the nursing students correct use of a conflict resolution skill (the two-challenge rule) in a high-fidelity simulation scenario, and b) satisfaction and self-confidence in learning (SSL).

Theoretical Framework

This study used Kolb’s Experiential Learning Theory (ELT) that presents a method of structuring the high-fidelity simulation using a learning cycle [12]. The learning cycle consisted of four phases: a) the concrete experience where the students participated in a high-fidelity simulation scenario, b) reflective observation whereby students gathered and organized information during the debriefing session, c) abstract conceptualization in which students considered what may have been done differently, and d) active experimentation, where students utilized what was learned to direct future practice.

This study also followed the revised Medical Research Council (MRC) framework for the development and evaluation of simulation interventions for RCTs [13,14]. The first two phases (Cycles A and B) were applied to the design and testing of the pre-simulation activities; the video simulation case study [15], and the paper case study, including the high-fidelity simulation scenario [16], before inclusion in this study. Both pre-simulation activities included scenarios where the student nurse had to address an information conflict and utilize a conflict management skill, the two-challenge rule. Applying progressive frameworks (e.g., the revised MRC) in simulation design and feasibility testing has value before conducting experimental studies (Cycle C) that can be time-consuming and costly.

Objectives

To evaluate the use of the conflict resolution skill (the two-challenge rule) by third-year undergraduate nursing students during the high-fidelity simulation scenario. To evaluate the nursing students’ evaluations of the entire simulation intervention related to satisfaction and self-confidence in learning (SSL).

## Materials and methods

Ethical Considerations

Approval for the study was obtained from the Research Ethics Committee of a large, urban Canadian university before nursing student recruitment and data collection commenced. Participation in this study was not part of a course grade. Additionally, the research assistants (RAs) and faculty members involved in the study did not have contact with the students previously, i.e., as a course instructor.

RCT design

**Figure 1:**
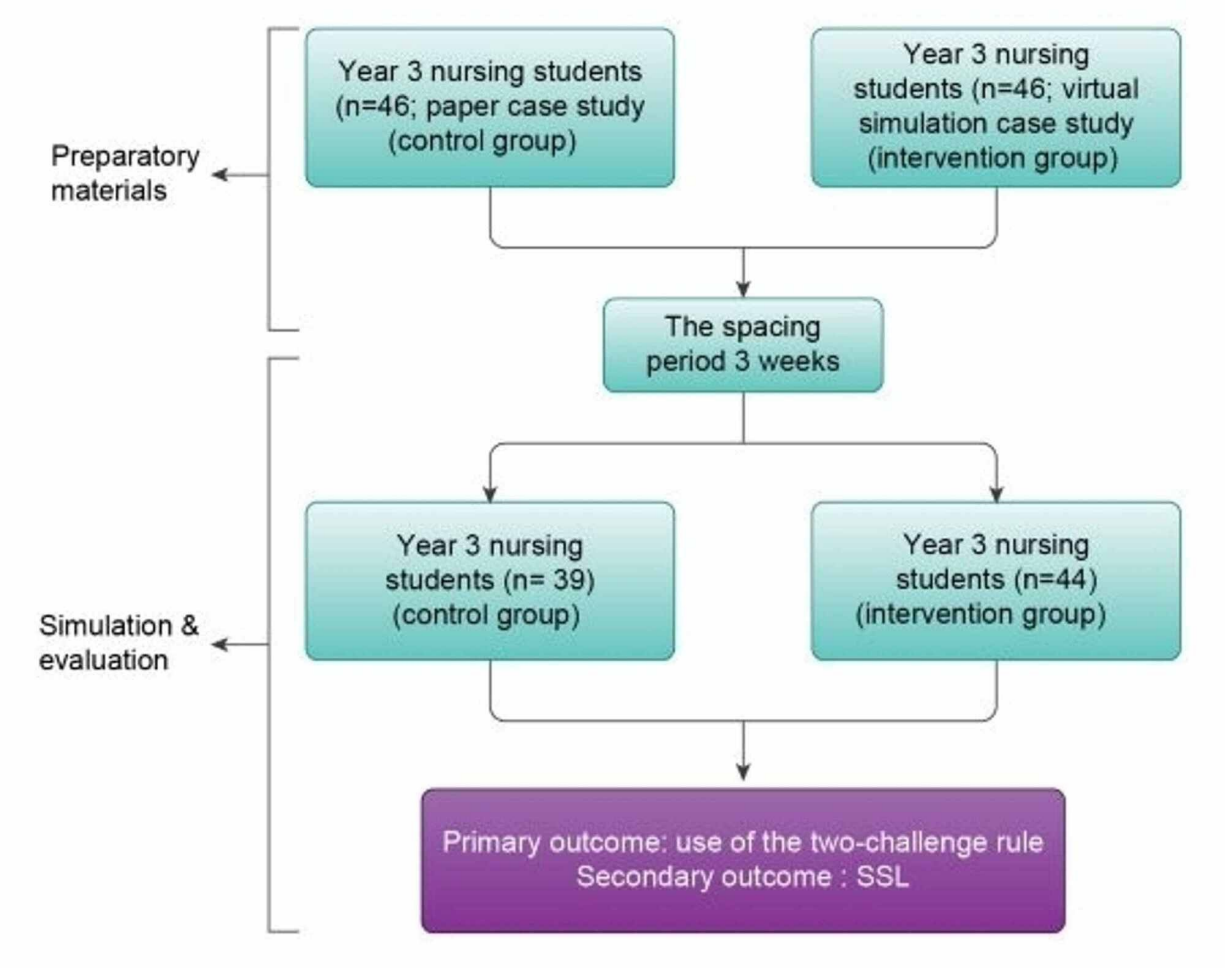
Study Schema

With the permission of the course professors, the RAs delivered in-class recruitment presentations during week two of the semester for year three nursing students. Upon receiving the students’ completed consent form and demographic questionnaire, an invitation was e-mailed to confirm receipt and advise them about their date to participate in the simulation. Students who consented to participate were randomized to the control group (n=46), where they received the formal paper case study, or the intervention group (n=46), where they received the interactive video simulation case study via e-mail. Both groups were invited to return and participate in a high-fidelity simulation scenario with a conflict management learning event (CMLE) followed by a debriefing session at the Simulation Center of a large urban University in Canada. Students were advised not to discuss the preparatory activities they received. See Figure 1 for the study schema.

The study sample size was determined using G*Power 3, a type I error of 0.05, a statistical power of 0.90, an effect size of d=0.80, with a total of 68 students (34 per group) required. Students were allocated to each group through a simple random allocation using IBM SPSS Statistics version 24.

Setting and Timing

Three weeks before their participation in the high-fidelity simulation scenario, nursing students in the control group were e-mailed the paper case study, including a modified version of the online TeamSTEPPS Mutual Support PowerPoint, reviewing the two-challenge rule. The nursing students randomized into the intervention group were e-mailed a link to the interactive video simulation case study [15], depicting a CMLE with a team member, where the two-challenge rule was utilized. The two-challenge rule pairs an advocacy statement (asserting one’s observation) and inquiry (request for the other’s reasoning). The video simulation case study had multiple-choice questions and decision points whereby the students could practice the two-challenge rule. The e-mail also contained an overview of the high-fidelity simulation scenario that all students, in both groups, were required to review and included information detailing the patient’s diagnosis, past medical history, medications, and treatment interventions. Other readings were about proper medication administration, i.e., how to administer an intramuscular (IM) injection.

The CMLE in the high-fidelity simulation scenario involved challenging a nurse about her incorrect mapping of an IM injection site; however, it was not revealed to the students. The e-mail instructed students to read the materials and complete the paper case study or the video simulation case study in advance of their participation in the high-fidelity simulation scenario. A printed checklist indicating the students completed the preparatory activities were required. Students handed their signed checklist to the RA on the date of participation in the high-fidelity simulation. The RAs were not blinded to student assignment because they did not assess the students’ use of the two-challenge rule. Students were also oriented to the high-fidelity simulation room a few minutes before participating in the simulation scenario to acquaint themselves with the human patient simulator (HPS) and the equipment.

Outline of high-fidelity simulation scenario

The primary learning objective specific to the simulation scenario was that third-year nursing students would demonstrate the effective use of the conflict management skill (the two-challenge rule) when communicating with the nurse to handle the information conflict. In the high-fidelity simulation scenario, there were three roles: the HPS, the third-year nursing student, and the Simulated Person (SP) (who played the role of a senior nurse). The scenario required the students to administer the patient’s medications; first, an oral diuretic followed by an IM injection. Next, the CMLE was initiated by the SP, and the student had to manage both the information conflict and safe medication administration simultaneously. Specifically, we focused on the students’ knowledge about the correct IM landmarking technique and the use of the two-challenge rule. The SP was formally trained in the Simulated Person Methodology Program at the University.

 Learning objectives related to the high-fidelity simulation scenario were cross-referenced by examining the course syllabi of the second year Clinical and Pathophysiology courses. This ensured that third-year students had the necessary baseline clinical knowledge related to both the assessment of and care for a patient with congestive heart failure and the process of medication administration.

Participating in the high-fidelity simulation

Third-year nursing students in the experimental and control groups participated in the high-fidelity simulation scenario on an individual basis over six weeks. They were expected to arrive at the Simulated Centre 15 minutes before their scheduled simulation scenario session. Students remained in a private room to prevent them from discussing their simulation experience with others and were advised not to share information. The educator briefly orientated them to the HPS, simulated patient room, the patient’s past medical history, and diagnosis, and then given an updated nursing report. A broad overview of the learning objectives for the high-fidelity simulation scenario, identical to the video simulation and paper case studies, was presented to them. After the pre-simulation briefing, the students were informed that they would be prompted when the high-fidelity simulation scenario began. 

Once the scenario was running, each student (n=83) was required to administer the patient’s medication and question the incorrect information that the senior nurse was providing regarding the landmarking of the IM injection. The students also had to vocalize their concerns a second time to ensure that the senior nurse heard them. Because team members have the responsibility to challenge a course of action that they believe might put a patient at risk, the student’s use of the two-challenge rule to manage the conflict was appropriate. If there were two challenges, the senior nurse took corrective action and allowed the student time to review the technique for administering IM injections. Once the conflict was resolved, the senior nurse left the simulated patient’s room.

Post-Scenario debriefing session

The debriefing session was conducted immediately after the students engaged in the high fidelity simulation scenario and was led by an educator who had extensive experience and training related to debriefing; moreover, was certified in Debriefing Assessment for Simulation in Healthcare (DASH). The Advocacy and Inquiry conversational approach [17] and the delta/plus model of inquiry [18] were utilized to discuss performance gaps. The in-person debriefing session aimed to help each student reconstruct and build on their existing knowledge to form mental representations of their performance through pattern recognition and cognitive inference. An educator’s overview that outlined the high-fidelity simulation scenario algorithm and the learning objectives helped guide the debriefing session. Some standard, open-ended questions that the educator asked during the debriefing session were: “How do you think you handled the conflict with the nurse?” and “Would you do anything differently the next time to manage and resolve the conflict?”

Data Collection

Objective 1: Utilization of the Two-Challenge Rule By Nursing Students

Two assessors observed the high-fidelity simulation scenario to evaluate the students’ accuracy and the frequency with which the two-challenge rule was applied during the simulation scenario. Both assessors were blinded to student allocation to either the intervention or control groups. The two-challenge rule was used correctly if the students paired advocacy (stating their observation) and inquiry (request for the other’s reasoning). The assessors both viewed the first five simulations collaboratively to estimate intercoder reliability.

Objective 2: Assessing the High-Fidelity Simulation

A survey instrument was administered to the third-year nursing students: the SSL, student version [19] Third-year students were asked to complete the instrument following the in-person debriefing session. The SSL is a 13-item instrument designed to measure participant satisfaction with the simulation activity and self-confidence in learning (8 items), using a 5-point measurement scale. Instrument reliability for the SSL has been tested using Cronbach’s alpha; the score for the satisfaction items was found to be 0.94, while the score for the self-confidence items was found to be 0.87 [20]. Of the 13 items on the SSL, five items address satisfaction with the teaching materials used in the simulation. In comparison, eight self-confidence items address participants’ feelings about their mastery of the learning content.

Attrition rate

To estimate attrition, the RA maintained a record of the number of third-year students who withdrew from the study before or at any time during their participation in the entire simulation intervention. Students who withdrew from the study were contacted via e-mail and asked to participate in a brief online survey administered through SurveyMonkey, including a brief closed-ended question followed by an open-ended clarification question about their reason for not participating.

Compliance of the educator with the simulation protocols

The educator completed the Compliance Data Collection Forms after each simulation session, recording whether intervention protocols were correctly followed and implemented for each student.

Data Analysis

We summarized participant characteristics using descriptive statistics, with continuous variables by median and Inter-quartile range (IQR= 75th percentile -25th percentile) and categorical variables by frequency and proportions. The participation rate of Year 3 students was determined by calculating the number of students who were invited and agreed to participate out of the total number eligible to participate. To achieve the primary objective, the chi-square test was performed to compare the proportion of those students who used two challenge-rule in the control and intervention groups.

Regarding our secondary objective, we utilized a Wilcoxon-Mann-Whitney test to compare the median of Student Satisfaction and Self-Confidence in Learning Scale as well as the total SSL score between control and intervention groups. In this study, Cronbach’s alpha coefficient and total item correlation analysis were used to determine the internal reliability and consistency of the scales within the context of reliability analysis. The data were analyzed in SPSS for Windows version 25.0.

## Results

Demographics

Overall, out of the eligible population of approximately N=180 third-year students, 83 (46.11%) students agreed to participate in this study. The control group comprised 39 students, and the intervention group included 44, all of whom completed the entire simulation intervention (see Figure 1). The majority of participants were female 74 (89.2%), and only 9 (10.8%) were male. The students’ age ranged from 22-26 years, with a median of 23 years (IQR=1).

Objective 1: Use of the Two-Challenge Rule

Two assessors observed the high-fidelity simulation scenario to evaluate the students’ accuracy and the frequency with which the two-challenge rule was applied during the simulation scenario. Both assessors were blinded to student allocation to either the intervention or control groups. The two-challenge rule was used correctly if the students paired advocacy (stating their observation) and inquiry (request for the other’s reasoning). The assessors both viewed the first five simulations independently to decipher if the student used the two-challenge rule correctly. They agreed that the student did or did not use the rule in the first five simulations (100% agreement).

To achieve the primary objective, we conducted a Chi-square test, which revealed a significant difference χ1 2,=16.4, p <.001 in the distribution of the two-challenge rule in the control and intervention groups. Table 1 shows the results from the Chi-square test and the proportions of the two-challenge rule in each group. We observed that 82% of students in the intervention group and 39% of students in the control group utilized the two-challenge rule.

**Table 1:** Comparison of using the two-challenge rule proportion

		Two-challenge rule		χ_df_^2^, p-value
		No	Yes	χ_1_^2^=16.4 , <.001
Group	Control	24 (61.5%)	15 (38.5%)	
	Intervention	8 (18.2%)	36 (81.8%)	

Objective 2: Student Satisfaction and Self-Confidence in Learning Scale

Results from the two independent-samples Wilcoxon-Mann-Whitney test showed a significant difference in the median of the total score of the SSL ( W=2.5, p <0.001), satisfaction (W =6.0, p<0.001), and self-confidence( W=68, p<0.001) in learning between third-year students in the control and intervention groups. Namely, students in the intervention group who completed the interactive video simulation case study had higher satisfaction and greater levels of self-confidence compared to those who received the paper case study. Table 2 summarizes the comparison of the two groups for both scales.

**Table 2:** Comparison of the SSL median scores SSL- satisfaction and self-confidence in learning

Scale	Group	Median	IQR	W	P-value
Total SSL score	Control	43.0	3.0	2.5	0.001
	Intervention	55.0	2.25		
Satisfaction	Control	16.0	3.5	6.0	0.001
	Intervention	22.0	2.0		
Self-confidence	Control	27.0	3.5	68.0	0.001
	Intervention	32.0	2.25		

Attrition rate

Students who withdrew from the study reported that they did not attend either due to a) an increased academic workload or b) personal or family issues. Students suggested that simulation activities should be conducted during weeks when there are fewer tests and assignments.

Compliance With Simulation Protocols

Responses to the open-ended question on the Compliance Data Collection Forms were categorized. The prevalent issue was that simulations were running late on several days due to some students commuting from their clinical placement to the Simulation Center at the University.

## Discussion

A pre-simulation activity using a previously refined video simulation case study enhanced preparation before an in-person, high-fidelity simulation, compared to that of the paper case study. The video simulation case study included interactive strategies to engage the students in conflict management with another team member. Students encountered decision-points by answering multiple-choice questions and receiving a summative score; moreover, they could repeatedly practice the communication skill [15]. Third-year students utilized the two-challenge rule significantly more than the control group and were more satisfied and self-confident after the high-fidelity simulation. A systematic review (SR) by Tyerman et al. (2016) examined pre-instructional activities that prepare students for a simulation experience. The SR results of the experimental studies that compared learner outcomes exposed to traditional pre-simulation activities (i.e., assigned readings) with alternative ones (i.e., video expert role-modelling) were mixed. However, the SR results suggested that alternative methods of pre-simulation preparation did impact positively on learning outcomes, knowledge, skills acquisition, and satisfaction compared to either traditional approaches or no preparation [11].

Interactive pre-simulation activities such as video simulation case studies and other types of virtual simulations are burgeoning. Future studies should prioritize curricular integration of more interactive pre-simulation preparatory activities and evaluate the impact on student learning, before in-person simulations or participation in virtual simulations [21-25]. Additionally, more experimental research is required that focuses on the pre-encounter learning aspect as simulations are being used for high stakes assessments and to replace clinical practice time. Currently, there are no best practice guidelines to inform the pre-simulation preparatory aspect, i.e., how to use and create various media to demonstrate learning (assessment).

This study also provided an example of how the revised MRC framework was applied to programmatically guide the design and feasibility testing of the simulation components before conducting this experimental study [13]. Simulation interventions encompass several parts that may act both dependently and independently and impact on learning outcomes. Consequently, it is essential to develop and refine simulations to ensure that issues of feasibility (e.g., recruitment, retention, acceptability) are favourable, before advancing to experimental studies. There are minimal examples in simulation education research where the revised MRC framework has been applied [13,15]. However, the relevance of adapting interventions to an MRC framework (2000, 2008) is commonly used in the public health discipline.

Our findings also re-emphasize the importance of using an established theoretical foundation to guide the design and implementation of simulations. According to evidence in previous systematic reviews, the use of theory in simulation intervention development has been inadequate [26,27]. Therefore, it can be challenging to gauge the extent to which theory contributes to our understanding of simulations and learning outcomes.

Lastly, understanding the effectiveness of educating undergraduate nursing students in conflict management via simulations is limited, but this study provides insight that an interactive activity is more effective at preparing students before an in-person simulation experience [9,28]. Research has also indicated that introducing conflict management training in a four-year baccalaureate program provides students with early opportunities to “practice” conflict resolution skills and familiarize themselves with conflict management events [28]. Opportunities to reinforce that learning throughout the program help students build confidence to handle subsequent conflicts in the clinical domain with nurses and preceptors [9,28]. Previous research has suggested that effective communication and collaboration reduces morbidity and mortality rates, the cost of care, and errors but improves job satisfaction and the retention of nursing staff [29].

Limitations

There were several limitations to this study. The sample was mostly female participants, yet nursing programs typically include a more diverse group of students. This study was conducted with undergraduate nursing students, limiting the generalizability of findings to other health care professionals and students. Another limitation was that the pre-briefing session, i.e., orientation to the simulation environment and simulation scenario, was not evaluated regarding learner outcomes. Nielson and Harder (2013) state that the pre-briefing activities may diminish student anxiety, stimulating student engagement, critical thinking, and reflective practice in a simulation [30]. Lastly, although we advised students not to discuss the preparatory activities and high-fidelity simulation experience with others, we could not control this outside of the simulation center.

## Conclusions

In light of this study’s findings and the broader implications, we conclude by emphasizing the importance of designing and refining simulation interventions before conducting experimental studies, and the importance of teaching undergraduate nursing students conflict management skills, i.e., the two-challenge rule. These two key findings advance our knowledge for how simulations can be programmatically designed, implemented, and evaluated to ensure high quality. Secondly, interactive pre-simulation activities may involve the completion of a video simulation case study or serious virtual game before the simulation experience. There is a need for more experimental studies to evaluate the optimal dosage and types of pre-simulation activity on learning outcomes. Lastly, as challenges in clinical practice intensify, it is increasingly crucial for nursing students to communicate effectively across hierarchies and resolve conflicts in teams to provide safe patient care.
